# Impact of opioid agonist therapies on non-fatal and fatal overdose: treatment patterns among individuals with opioid use disorder in Ontario, Canada

**DOI:** 10.3389/fpubh.2026.1894981

**Published:** 2026-09-29

**Authors:** Kenneth Lee, Vincent Lam, Jennifer Nicolle, Martine Drolet, Maher Abdel-Sattar, Ginnie Ng, Melissa Snider-Adler

**Affiliations:** 1 London RAAM Clinic, London, ON, Canada; 2 Departments of Psychiatry and Family Medicine, Western University, London, ON, Canada; 3 Coderix Medical Clinic, Toronto, ON, Canada; 4 Department of Family and Community Medicine, University of Toronto, Toronto, ON, Canada; 5 Centre for Addiction and Mental Health, Toronto, ON, Canada; 6 Indivior Inc., Montreal, QC, Canada; 7 Indivior Inc., North Chesterfield, VA, United States; 8 IQVIA Solutions Canada Inc., Kirkland, QC, Canada; 9 South Oshawa Clinic, Oshawa, ON, Canada

**Keywords:** addiction, buprenorphine, fentanyl, opioids, overdose, substance use disorders

## Abstract

**Background:**

Although opioid agonist therapies (OAT) including buprenorphine extended-release (BUP-XR), transmucosal buprenorphine (TM-BUP), methadone and slow-release oral morphine (SROM) are effective and established medications for opioid use disorder (MOUD) available in Canada, differences in opioid-related overdose (OD) risk reduction across OAT types and patterns of treatment use are not well characterized.

**Objective:**

To assess the impact of OAT on opioid-related non-fatal and fatal OD among individuals with OUD in Ontario, Canada, and to examine patterns of OAT use.

**Methods:**

A retrospective, population-based, nested case-control study was conducted using ICES administrative health data. Adults with opioid use disorder (OUD) who were administered ≥1 OAT from January 2022 through June 2024 were included. Cases with opioid-related non-fatal or fatal OD were matched 1:10 to controls on age, sex, and follow-up duration. Adjusted odds ratios were estimated using weighted exact conditional logistic regression.

**Results:**

Among 45,243 individuals with OUD who started OAT, 4,109 (9.1%) experienced a non-fatal OD and 770 (1.7%) had a fatal OD. Fatal OD cases were most commonly aged 35–49 years; non-fatal cases were most prevalent among those aged 18–34 years. Most OD cases were male and resided in urban areas. Substance use disorders other than OUD and anxiety disorders were more prevalent among OD cases than controls. Of non-fatal and fatal OD cases, 75.9% and 84.9% were not covered by an OAT for at least 80% of the time between cohort entry and index OD event, respectively. Of all OATs, BUP-XR had the lowest proportion of patients with non-fatal and fatal OD compared to no treatment. Both BUP-XR and TM-BUP were associated with significantly lower odds of non-fatal and fatal OD compared to no OAT, while methadone and SROM did not show similar improvements.

**Conclusion:**

OAT type and treatment continuity were associated with different OD risk reductions. Supporting sustained OAT engagement and expanding access to buprenorphine, including BUP-XR, may reduce non-fatal and fatal OD risk among individuals with OUD. Limitations include potential misclassification in administrative data, incomplete or inaccurate capture of opioid-related OD events and OAT use, and absence of a direct measure of OAT adherence.

## 1 Introduction

Opioid use disorder (OUD) is a chronic, relapsing condition marked by compulsive opioid use, tolerance, and withdrawal symptoms, leading to significant distress or functional impairment (1), and posing substantial challenges for impacted individuals (2) and health systems (3). Opioid overdose (OD) is a leading cause of preventable death, with Canada experiencing persistently high rates of opioid-related fatalities (4–8). Between January 2016 and December 2025, 56,631 apparent opioid toxicity deaths were recorded nationally in Canada, with fentanyl implicated in 56% of cases and non-pharmaceutical opioids involved in 82% (6). In response to this ongoing public health crisis, opioid agonist therapies (OATs), including methadone and buprenorphine, have become widely adopted and are recognized as effective, evidence-based medications for opioid use disorder (MOUD) (9).

Extensive research demonstrates that OAT significantly reduces the risk of opioid-related harms, including hospitalizations and fatal ODs (10–12). Accordingly, Canadian OAT prescribing guidelines highlight key treatment considerations, including modality, intensity, duration, and dosing frequency, to tailor therapy to individual patient needs (13). The latest Canadian guidelines were updated in 2024 to recommend both buprenorphine and methadone as first-line therapies, and slow-release oral morphine (SROM) as a second-line therapy (14). Over time, OAT options have evolved to better align with patient preferences and clinical judgment, offering a broader range of formulations, including both daily and long-acting monthly formulations (15), thereby increasing flexibility in treatment delivery.

Despite these advances, challenges remain across different OAT modalities and stages of treatment. Evidence suggests a higher risk of treatment attrition during the first month following initiation among patients starting transmucosal buprenorphine (TM-BUP), as well as an elevated risk of mortality during the initial month of methadone treatment compared with later treatment periods (16). These findings highlight the importance of understanding how odds of OD vary by treatment type and timing.

As such, further investigation is needed to characterize the risk of opioid-related OD among various OAT formulations using large, population-based studies. Improved understanding of these dynamics has the potential to inform clinical decision-making, refine treatment guidelines, and optimize patient outcomes. The objective of this study was to provide a comprehensive assessment of the impact of OATs on opioid-related non-fatal and fatal ODs among individuals with OUD in Ontario, Canada.

## 2 Materials and methods

### 2.1 Study design and data sources

A retrospective, population-based, nested case-control study was conducted to assess opioid-related ODs among adults with OUD in Ontario, Canada. The study used de-identified, record-level, encoded linkable administrative health data held at ICES, an independent, non-profit research institute formerly known as the Institute for Clinical and Evaluative Sciences. ICES data encompasses much of the publicly funded administrative health services records for the Ontario population eligible for universal health coverage since 1986 and integrates administrative data, registries, and surveys.

Databases used in this study included the Narcotics Monitoring System (NMS) to capture all prescription dispensations for controlled substances dispensed from community pharmacies in Ontario, regardless of payer. This study also used the Drug and Drug/Alcohol Related Death (DDARD) database to identify opioid-related deaths from the Ontario Office of the Chief Coroner, the Discharge Abstract Database (DAD) to capture inpatient hospitalizations, the National Ambulatory Care Reporting System (NACRS) to capture emergency department (ED) visits, the Ontario Mental Health Reporting System (OMHRS) to capture data on individuals receiving mental health services, the Ontario Health Insurance Plan (OHIP) Claims Database to capture physician billing claims and outpatient procedures, and the Ontario Drug Benefit (ODB) database to capture eligible prescription drug claims.

### 2.2 Study population

A base cohort was first identified, consisting of Ontario residents aged 18 years or older with OUD who received at least one prescription for OAT between January 1, 2022, and June 30, 2024 (the selection period). A lookback period extending from base cohort entry back to April 1, 2002 was used to assess eligibility criteria and demographics and clinical characteristics. Individuals were required to have no OAT prescriptions in the 12 months prior to base cohort entry. OUD was identified using ICD-9 or ICD-10-CA diagnosis codes for opioid use, dependence, or abuse (Supplementary Table 1). These codes were identified using the DAD, NACRS, OMHRS, and OHIP databases. OAT use was identified through claims for buprenorphine extended-release (BUP-XR), TM-BUP (both sublingual and buccal formulations), methadone, or SROM identified in the NMS database.

After base cohort entry, individuals were followed for up to 24 months to evaluate study outcomes (analysis period). Those who experienced a non-fatal or fatal OD during follow-up were classified as cases. Two case cohorts were created: (1) opioid-related fatal OD cases, defined by confirmed death in the DDARD database, and (2) opioid-related non-fatal OD cases, defined by an ICD-10-CA code for opioid toxicity recorded in ED visits and/or hospital discharge records. The index date was defined as the date of opioid-related OD event. Notably, the case cohorts were not mutually exclusive; individuals who experienced a non-fatal and subsequent fatal OD could be included in both cohorts. Individuals who experienced more than one non-fatal overdose were included only at the time of their first non-fatal overdose.

Each case was matched to 10 controls from the base cohort who did not experience a fatal or non-fatal opioid-related OD, on the basis of age (±3 years), sex, and time at risk of OD (i.e., time between cohort entry and the OD event of the matched case).

### 2.3 Exposure

All prescription dispensations for OATs indicated for OUD between the Cohort Entry and the index date were identified in the NMS dataset using relevant drug identification numbers (DINs). OAT use at index (i.e., time of opioid-related OD event) was captured using DINs and described at the product-level. The duration of sustained use of the index OAT was defined as the length of continuous use of the OAT on the index date, measured from the start date of the OAT dispensation to the index date. Patients with OAT coverage at index (i.e., have discontinued temporarily or permanently within the study period) were classified as having “No OAT” at index. Patients with overlapping dispensations of different OATs were considered on “Multiple OATs” for the duration of the overlap.

Time on and off OAT was based on dispensing patterns throughout the study period. Each OAT dispensation was assigned a period of protective effect (“coverage”) beginning on the dispensation date, with the duration of coverage based on pharmacokinetic profiles from product monographs and established literature. Individuals were considered to be “on OAT” during periods covered by an active OAT dispensation. TM-BUP, methadone, and SROM had 1 day of protective effect assigned, whereas BUP-XR was assigned 1 month (30 days) of protective effect.

The OAT dose was calculated using the quantity and days supply recorded in the NMS database for each respective dispensation. Medication strength was determined according to the active ingredient value specified for each DIN. If multiple dispensations of the same OAT type were recorded on a single date, the total daily dose was obtained by summing the individual daily doses from each.

### 2.4 Outcomes

The number of opioid-related non-fatal and fatal OD cases overall and stratified by OAT use at the time of index OD event were reported. Patterns of OAT utilization were described, along with key demographic and clinical characteristics of individuals who initiated OAT, to identify factors potentially associated with opioid-related non-fatal and fatal ODs. The presence of illicit substances during index OD events was also reported. For fatal OD cases, substances were identified in the DDARD database at ICES, which contains records from coronial investigations. A substance could be coded as “attributed to death” or “detected, not attributed to death.” For non-fatal OD cases, poisonings were identified using ICD-10-CA codes.

### 2.5 Analysis

Descriptive statistics were used to summarize cohort characteristics. Categorical variables were reported as counts and percentages, and continuous variables were reported using means and standard deviations, medians and interquartile ranges, and minimum and maximum values.

Crude and adjusted odds ratios (ORs) with 95% confidence intervals (CIs) for opioid-related non-fatal and fatal OD associated with OAT use at index were estimated using conditional logistic regression. Adjusted models included OAT use at index, rurality, comorbidities of interest (hepatitis C, schizophrenia, mood disorders, and anxiety disorders), prior non-fatal OD, and time spent on OAT.

All analyses were conducted in accordance with ICES standard operating procedures, including data quality checks and privacy policies (e.g., results based on less than 6 patients were not disclosed). Statistical analyses were performed using SAS Enterprise Guide (SAS Institute, Cary, NC), version 9.4 or higher.

## 3 Results

### 3.1 Patient demographic and clinical characteristics

In total, 45,243 patients in Ontario were included in the cohort. Among them, 4,109 (9.1%) had at least one non-fatal OD event and 770 (1.7%) had a fatal OD event (Table 1).

**Table 1:** Demographic and clinical characteristics at base cohort entry date.

Demographic characteristics	Variable	Base cohort	Opioid-related non-fatal OD cases	Opioid-related non-fatal OD controls	Opioid-related fatal OD cases	Opioid-related fatal OD controls
	Sample size	*N* = 45,243	*N* = 4,109	*N* = 41,090	*N* = 770	*N* = 7,700
Follow-up time (days)	Mean (SD)	493.30 (238.23)	254.06 (196.99)	254.06 (196.96)	281.63 (194.79)	281.63 (194.68)
	Median (Q1-Q3)	561 (291–730)	216 (80–400)	216 (80–400)	252 (116–421)	252 (116–421)
Age (years)	Mean (SD)	41.60 (12.82)	36.99 (10.47)	37.10 (10.33)	39.54 (11.44)	39.58 (11.29)
	Median (Q1-Q3)	39 (32–50)	35 (30–43)	35 (30–43)	38 (31–47)	38 (31–47)
Age group (years)	18–34 - *n* (%)	15,409 (34.1%)	1,917 (46.7%)	19,166 (46.6%)	298 (38.7%)	2,918 (37.9%)
	35–49 - *n* (%)	18,228 (40.3%)	1,648 (40.1%)	16,515 (40.2%)	316 (41.0%)	3,265 (42.4%)
	50–64 - *n* (%)	9,006 (19.9%)	487 (11.9%)	4,871 (11.9%)	142 (18.4%)	1,363 (17.7%)
	65+ - *n* (%)	2,600 (5.7%)	57 (1.4%)	538 (1.3%)	14 (1.8%)	154 (2.0%)
Sex, male	Male - *n* (%)	28,556 (63.1%)	2,780 (67.7%)	27,800 (67.7%)	538 (69.9%)	5,380 (69.9%)
Rurality, urban (population >10,000)	Urban - *n* (%)	38,154 (84.3%)	3,670 (89.3%)	34,258 (83.4%)	687 (89.2%)	6,548 (85.0%)
Neighborhood income quintile	Q1 (lowest) - *n* (%)	17,626 (39.0%)	1,907 (46.4%)	15,917 (38.7%)	318 (41.3%)	3,041 (39.5%)
	Q2 - *n* (%)	10,099 (22.3%)	910 (22.1%)	9,185 (22.4%)	197 (25.6%)	1,712 (22.2%)
	Q3 - *n* (%)	7,420 (16.4%)	582 (14.2%)	6,876 (16.7%)	107 (13.9%)	1,240 (16.1%)
	Q4 - *n* (%)	5,558 (12.3%)	384 (9.3%)	5,065 (12.3%)	75 (9.7%)	984 (12.8%)
	Q5 (highest) - *n* (%)	4,540 (10.0%)	326 (7.9%)	4,047 (9.8%)	73 (9.5%)	723 (9.4%)
ED visit (opioid toxicity)^†^	*n* (%)	3,981 (8.8%)	3,917 (95.3%)	0 (0.0%)	195 (25.3%)	445 (5.8%)
	Mean (SD)	0.15 (0.68)	0.95 (0.21)	0.00 (0.00)	0.44 (1.20)	0.09 (0.45)
Non-fatal overdose(s)^†^, any	*n* (%)	4,109 (9.1%)	NA	NA	173 (22.5%)	462 (6.0%)

ED: emergency department; OD: overdose; SD: standard deviation; NA: not applicable. ^†^ Count of individuals with ED visits and non-fatal ODs between the cohort entry date and OD event or censoring (death, loss to follow-up, or end of analysis period; whichever is first).

Non-fatal OD cases were primarily aged 18–34 years (46.7%), whereas fatal OD cases were most often aged 35–49 years (41.0%) (Table 1). The majority of both case groups were male (non-fatal OD: 67.7%; fatal OD: 69.9%) and resided in urban settings (non-fatal OD: 89.3%; fatal OD: 89.2%) (Table 1). OD cases were disproportionately concentrated within the lowest neighborhood income quintile, accounting for 46.4% of non-fatal OD and 41.3% of fatal cases (Table 1).

Since non-fatal ODs were identified from hospitalization and ED records, 95.3% of non-fatal OD cases had an ED record of opioid toxicity. These ED visits may represent the index event used to define non-fatal OD cases. In addition, 25.3% of fatal OD cases had at least one prior ED visit for opioid toxicity (Table 1).

Comorbid substance use disorders other than OUD were the most prevalent clinical conditions among cases, reported in 80.2% of non-fatal OD cases vs. 56.0% of controls, and 75.3% of fatal OD cases vs. 56.1% of controls (Table 2). Anxiety disorders were also common, affecting 54.3% of non-fatal OD cases and 48.6% of fatal OD cases, compared with 38.5 and 38.2% of controls, respectively (Table 2). Comorbid mood disorders were observed in 39.6% of non-fatal OD cases and 35.2% of fatal OD cases, vs. 26.7% and 27.6% of controls, respectively (Table 2). Schizophrenia was present in 24.2% of non-fatal OD cases and 20.5% of fatal OD cases, compared with 11.1% and 12.1% of controls, respectively (Table 2).

**Table 2:** Comorbidities at base cohort entry date.

Comorbidity history^†^	Base cohort, *n* (%)	Opioid-related non-fatal OD cases, *n* (%)	Opioid-related non-fatal OD controls, *n* (%)	Opioid-related fatal OD cases, *n* (%)	Opioid-related fatal OD controls, *n* (%)
	*N* = 45,243	*N* = 4,109	*N* = 41,090	*N* = 770	*N* = 7,700
No comorbidities	10,123 (22.4%)	373 (9.1%)	9,640 (23.5%)	85 (11.0%)	1,783 (23.2%)
HIV	439 (1.0%)	81 (2.0%)	307 (0.7%)	15 (1.9%)	68 (0.9%)
Hepatitis C	3,577 (7.9%)	650 (15.8%)	2,721 (6.6%)	110 (14.3%)	620 (8.1%)
Substance use disorders aside from OUD	24,975 (55.2%)	3,296 (80.2%)	23,022 (56.0%)	580 (75.3%)	4,322 (56.1%)
Psychiatric disorders					
Organic dissociative disorder	0 (0.0%)	0 (0.0%)	0 (0.0%)	0 (0.0%)	0 (0.0%)
Schizophrenia	5,318 (11.8%)	993 (24.2%)	4,581 (11.1%)	158 (20.5%)	931 (12.1%)
Mood disorders	12,750 (28.2%)	1,628 (39.6%)	10,988 (26.7%)	271 (35.2%)	2,126 (27.6%)
Anxiety disorders	17,626 (39.0%)	2,230 (54.3%)	15,833 (38.5%)	374 (48.6%)	2,940 (38.2%)
PTSD	1,633 (3.6%)	254 (6.2%)	1,356 (3.3%)	39 (5.1%)	272 (3.5%)
COPD	5,759 (12.7%)	351 (8.5%)	2,893 (7.0%)	101 (13.1%)	786 (10.2%)
Asthma	11,575 (25.6%)	1,137 (27.7%)	10,760 (26.2%)	228 (29.6%)	1,955 (25.4%)
Liver cirrhosis	1,216 (2.7%)	106 (2.6%)	677 (1.6%)	30 (3.9%)	185 (2.4%)

COPD: chronic obstructive pulmonary disease; HIV: human immunodeficiency virus; OD: overdose; OUD: opioid use disorder; PTSD: post-traumatic stress disorder. ^†^ See Supplementary File for details on how comorbidities were defined.

Compared with matched controls, a greater proportion of non-fatal and fatal OD cases had a history of medications that may increase OD risk, including gamma-aminobutyric acid (GABA) agonists, drugs known to prolong the QT interval, serotonergic agents, and benzodiazepines (Table 3).

**Table 3:** Medication history in the 6 months prior to index overdose date.

Medication history	Opioid-related non-fatal OD cases, *n* (%)	Opioid-related non-fatal OD controls, *n* (%)	Opioid-related fatal OD cases, *n* (%)	Opioid-related fatal OD controls, *n* (%)
	*N* = 4,109	*N* = 41,090	*N* = 770	*N* = 7,700
GABA agonists	1,000 (24.3%)	7,478 (18.2%)	212 (27.5%)	1,574 (20.4%)
Drugs that can prolong QT interval	557 (13.6%)	3,680 (9.0%)	115 (14.9%)	746 (9.7%)
Drugs that affect electrolytes	46 (1.1%)	260 (0.6%)	6 (0.8%)	50 (0.6%)
Serotonergic drugs	714 (17.4%)	5,998 (14.6%)	140 (18.2%)	1,212 (15.7%)
NNRTIs	0 (0.0%)	7 (0.0%)	0 (0.0%)	0 (0.0%)
CYP3A4 inducers	34 (0.8%)	157 (0.4%)	1–5*	30 (0.4%)
Benzodiazepines	726 (17.7%)	6,096 (14.8%)	159 (20.6%)	1,258 (16.3%)

GABA: gamma-aminobutyric acid; OD: overdose; NNRTIs: non-nucleoside reverse transcriptase inhibitors. ^*^Denotes ranges resulting from ICES privacy policies for cell counts <6. Other counts may also not be disclosed to prevent back-calculation.

### 3.2 Patterns of OAT use

Trends in OAT use at the time of OD event were similar between case cohorts. The majority of non-fatal (64.8%) and fatal (83.1%) OD cases were not covered by an OAT at the time of OD event (Table 4). Among non-fatal OD cases, BUP-XR was the least common OAT at the time of OD event (2.1%), followed by SROM (3.7%), TM-BUP (6.2%), multiple OATs (7.7%), and methadone (15.5%). Similarly, BUP-XR was the least common OAT at the time of OD event among fatal OD cases (≤0.7%), followed by multiple OATs (≤2.1%), SROM (2.3%), TM-BUP (4.8%), and methadone (7.5%) (Table 4).

**Table 4:** Patterns of OAT use at index overdose date.

Pattern of OAT	Variable value	Opioid-related non-fatal OD cases	Opioid-related non-fatal OD controls	Opioid-related fatal OD cases	Opioid-related fatal OD controls
	Sample size	*N* = 4,109	*N* = 41,090	*N* = 770	*N* = 7,700
Index OAT					
No OAT	*n* (%)	2,664 (64.8%)	19,647 (47.8%)	640 (83.1%)	3,786 (49.2%)
BUP-XR	*n* (%)	85 (2.1%)	1,353 (3.3%)	1–5*	252 (3.3%)
TM-BUP	*n* (%)	254 (6.2%)	10,269 (25.0%)	37 (4.8%)	1,874 (24.3%)
Methadone	*n* (%)	636 (15.5%)	6,803 (16.6%)	58 (7.5%)	1,187 (15.4%)
SROM	*n* (%)	152 (3.7%)	1,148 (2.8%)	18 (2.3%)	233 (3.0%)
Multiple OATs^†^	*n* (%)	318 (7.7%)	1,870 (4.6%)	12–16*	368 (4.8%)
Duration of sustained use of index OAT (days), by OAT type					
BUP-XR	Mean (SD)	39.35 (40.66)	49.89 (62.43)	*	54.35 (72.33)
	Median (Q1-Q3)	29 (13–49)	30 (15–59)	*	29 (17–68)
TM-BUP	Mean (SD)	40.35 (73.49)	75.29 (106.70)	36.54 (52.15)	87.50 (116.07)
	Median (Q1-Q3)	10 (4–43)	31 (9–94)	16 (6–39)	39 (13–114)
Methadone	Mean (SD)	22.16 (45.70)	28.37 (59.30)	22.24 (49.11)	31.13 (61.68)
	Median (Q1-Q3)	6 (3–22)	6 (3–27)	6 (3–14)	6 (3–30)
SROM	Mean (SD)	50.20 (79.51)	75.82 (104.31)	34.83 (51.10)	86.17 (115.22)
	Median (Q1-Q3)	18 (7–56)	35 (11–90)	17 (6–35)	36 (12–109)
Dose of index OAT, by OAT type^≠^					
BUP-XR	High dose (≥300 mg), *n* (%)	70 (82.4%)	932 (68.9%)	1–5*	167 (66.3%)
	Low dose (<300 mg), *n* (%)	15 (17.6%)	421 (31.1%)	0 (0.0%)	85 (33.7%)
TM-BUP	High dose (≥16 mg), *n* (%)	94 (37.0%)	3,417 (33.3%)	15 (40.5%)	657 (35.1%)
	Low dose (<16 mg), *n* (%)	160 (63.0%)	6,852 (66.7%)	22 (59.5%)	1,217 (64.9%)
Methadone	High dose (≥100 mg), *n* (%)	251 (39.5%)	3,194 (46.9%)	25 (43.1%)	597 (50.3%)
	Low dose (<100 mg), *n* (%)	385 (60.5%)	3,609 (53.1%)	33 (56.9%)	590 (49.7%)
SROM	High dose (≥800 mg), *n* (%)	24 (15.8%)	141 (12.3%)	0 (0.0%)	26 (11.2%)
	Low dose (<800 mg), *n* (%)	128 (84.2%)	1,007 (87.7%)	18 (100.0%)	207 (88.8%)
Proportion of total time on OAT	<0.80 - *n* (%)	3,120 (75.9%)	23,741 (57.8%)	654 (84.9%)	4,613 (59.9%)
	0.80–1.00 - *n* (%)	989 (24.1%)	17,349 (42.2%)	116 (15.1%)	3,087 (40.1%)

BUP-XR: buprenorphine extended-release; OAT: opioid agonist therapy; SD: standard deviation; SROM: slow-release oral morphine; TM-BUP: transmucosal buprenorphine. * Denotes ranges resulting from ICES privacy policies for cell counts <6. Other counts may also not be disclosed to prevent back-calculation. ^†^ See Supplementary File for details of multiple OAT use.^ ≠^ Reported doses included both initiation/induction and maintenance doses, where applicable.

Time on OAT differed between OD case cohorts and their matched controls. The majority of non-fatal (75.9%) and fatal (84.9%) OD cases spent less than 80% of the time between base cohort entry and index OD event on OAT (Table 4). In contrast, 57.8% and 59.9% of non-fatal and fatal OD controls spent at least 80% of the time on OAT, respectively (Table 4). Similarly, the mean duration of sustained OAT use at the time of OD event was often longer among non-fatal cases compared to fatal cases, and longer among controls compared to cases (Table 4).

Dosing patterns varied by OAT and case cohorts. Based on low-dose and high-dose definitions listed in Table 4, OD cases more often had low-dose regimens for TM-BUP (non-fatal: 63.0%; fatal: 59.5%), methadone (non-fatal: 60.5%; fatal: 56.9%), and SROM (non-fatal: 84.2%; fatal: 100.0%). Likely reflecting initiation dosing, a greater proportion of OD cases were on high-dose regimens (i.e., 300 mg) of BUP-XR (non-fatal: 82.4%; fatal: *not disclosed due to ICES privacy policies for counts <6*) given that the mean duration of BUP-XR continuous coverage prior to an OD event was approximately 30–50 days, equating to only 1 or 2 BUP-XR monthly injections prior to non-fatal or fatal OD occurrence (Table 4).

### 3.3 Impact of OAT on opioid-related non-fatal and fatal OD

Adjusted odds ratio (aOR) calculations showed that OATs were differentially associated with the odds of non-fatal and fatal OD compared with no OAT coverage at index OD event (Figures 1, 2). Both formulations of buprenorphine (BUP-XR and TM-BUP) were associated with significantly lower odds of non-fatal OD (BUP-XR: aOR = 0.72, 95% CI 0.59–0.87; TM-BUP: aOR = 0.36, 95% CI 0.32–0.40) and fatal OD (BUP-XR: aOR = 0.46, 95% CI 0.21–0.97; TM-BUP: aOR = 0.56, 95% CI 0.39–0.79) (Figures 1, 2). In contrast, SROM was associated with increased odds of both non-fatal OD (aOR = 1.43, 95% CI 1.23–1.67) and fatal OD (aOR = 1.58, 95% CI 1.01–2.50) relative to no OAT coverage at index OD event (Figures 1, 2). Methadone use showed no change in the odds of non-fatal OD (aOR = 1.08, 95% CI 0.99–1.18) or fatal OD (aOR = 1.08, 95% CI 0.80–1.46) compared with no OAT coverage at index OD event (Figures 1, 2).

**Figure 1:**
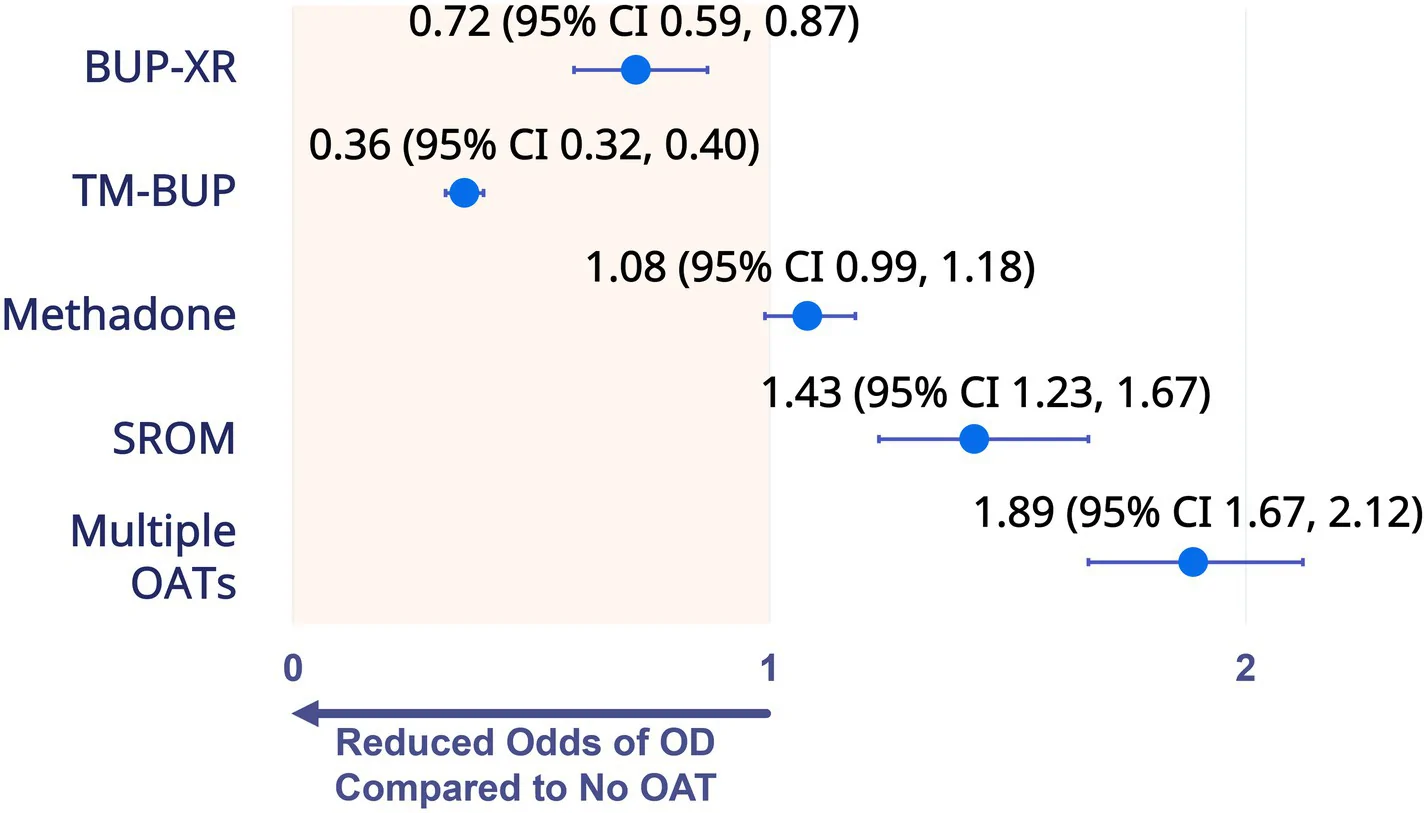
Effect of OATs on opioid-related non-fatal overdose. BUP-XR: buprenorphine extended-release; CI: confidence interval; OAT: opioid agonist therapy; OR: odds ratio; SROM: slow-release oral morphine; TM-BUP: transmucosal buprenorphine.

**Figure 2:**
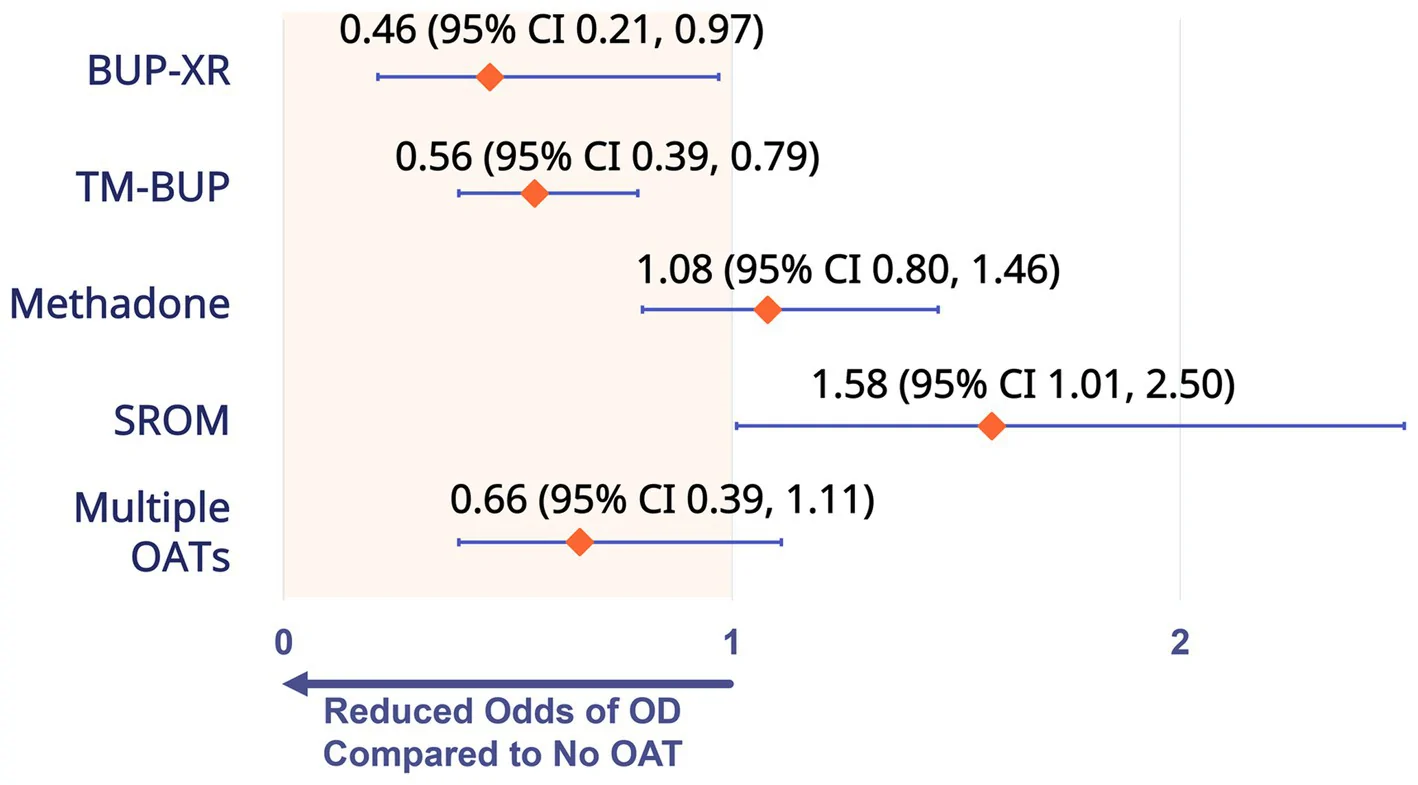
Effect of OATs on opioid-related fatal overdose. BUP-XR: buprenorphine extended-release; CI: confidence interval; OAT: opioid agonist therapy; OR: odds ratio; SROM: slow-release oral morphine; TM-BUP: transmucosal buprenorphine.

**Table 5:** Effect of OATs and other factors on opioid-related non-fatal overdose.

Variables	Adjusted OR	95% CI of adjusted OR
OAT use at index		
BUP-XR vs. no OAT	0.72	(0.59, 0.87)
TM-BUP vs. no OAT	0.36	(0.32, 0.4)
Methadone vs. no OAT	1.08	(0.99, 1.18)
SROM vs. no OAT	1.43	(1.23, 1.67)
Multiple OATs vs. no OAT	1.89	(1.69, 2.12)
Rurality		
Urban (population >10,000) vs. rural (population ≤10,000)	1.48	(1.33, 1.65)
Comorbidities^†^		
Hepatitis C (yes/no)	1.97	(1.79, 2.17)
Schizophrenia (yes/no)	1.81	(1.66, 1.98)
Mood disorders (yes/no)	1.23	(1.14, 1.33)
Anxiety disorders (yes/no)	1.44	(1.33, 1.55)
Total time on any/all OAT		
0.80–1.00 vs. <0.80	0.79	(0.75, 0.83)

BUP-XR: buprenorphine extended-release; CI: confidence interval; OAT: opioid agonist therapy; OR: odds ratio; SROM: slow-release oral morphine; TM-BUP: transmucosal buprenorphine^†^See Supplementary File for details on how comorbidities were defined.

**Table 6:** Effect of OATs and other factors on opioid-related fatal overdose.

Variables	Adjusted OR	95% CI of adjusted OR
OAT use at index		
BUP-XR vs. no OAT	0.46	(0.21, 0.97)
TM-BUP vs. no OAT	0.56	(0.39, 0.79)
Methadone vs. no OAT	1.08	(0.8, 1.46)
SROM vs. no OAT	1.58	(1.01, 2.5)
Multiple OATs vs. no OAT	0.66	(0.39, 1.11)
Rurality		
Urban (population >10,000) vs. rural (population ≤10,000)	1.31	(1.02, 1.68)
Comorbidities^†^		
Hepatitis C (yes/no)	1.40	(1.1, 1.79)
Schizophrenia (yes/no)	1.28	(1.03, 1.6)
Mood disorders (yes/no)	1.09	(0.9, 1.31)
Anxiety disorders (yes/no)	1.22	(1.02, 1.46)
Non-fatal overdose(s)		
Yes vs. no	3.71	(2.99, 4.61)
Total time on any/all OAT		
0.80–1.00 vs. <0.80	0.85	(0.74, 0.97)

BUP-XR: buprenorphine extended-release; CI: confidence interval; OAT: opioid agonist therapy; OR: odds ratio; SROM: slow-release oral morphine; TM-BUP: transmucosal buprenorphine^†^See Supplementary File for details on how comorbidities were defined.

Several demographic and clinical factors were also associated with the odds of having an OD event (Tables 5, 6). Residing in an urban area was associated with higher odds of both non-fatal OD and fatal OD compared with rural residence (Tables 5, 6). Comorbid hepatitis C, schizophrenia, and anxiety disorders were all associated with elevated odds of OD (Tables 5, 6). Mood disorders were trending to increased odds of fatal ODs and were significantly associated with greater odds of non-fatal OD (Tables 5, 6).

Finally, greater consistency of OAT use was associated with reduced odds of OD. High OAT coverage of 80–100% of the pre-index period (time between base cohort entry and index OD event) was associated with reduced odds of non-fatal OD (aOR = 0.79, 95% CI 0.75–0.83) and fatal OD (aOR = 0.85, 95% CI 0.74–0.97) compared with lower OAT coverage (Tables 5, 6).

### 3.4 Presence of illicit substances and drug misuse during OD event

Illicit substances, including non-pharmaceutical opioids and non-opioids, were often identified at the time of OD events. The full list of opioid and non-opioid illicit substances can be found in Supplementary Tables 8, 9. In non-fatal OD cases, opioid poisoning was most commonly associated with fentanyl and its analogs (61.3%). Other common substances at time of OD included narcotics and psychodysleptics/hallucinogens (intentional: 6.6%; undetermined intent: 12.3%; accidental: 79.8%), cocaine (5.5%), and methadone (4.3%) (Supplementary Table 8).

The majority of fatal OD events also involved non-opioids (directly contributed: 66.9%; detected: 40.3%) and non-pharmaceutical opioids (directly contributed: 89.6%; detected: 27.9%) (Supplementary Table 9). Specifically, the grouped category of fentanyl and fentanyl analogs (88.4%), cocaine (40.0%) and methamphetamine (37.1%) were among the most common substances that directly contributed to fatal events (Supplementary Table 9).

## 4 Discussion

This retrospective study examined opioid-related OD outcomes among patients with OUD in Ontario, with a focus on demographic characteristics, OAT type, and treatment coverage at the time of non-fatal and fatal OD events. OAT type and treatment continuity were associated with different risk of OD. Notably, individuals receiving BUP-XR and TM-BUP experienced a significantly lower risk of both non-fatal and fatal OD compared with individuals not covered by OAT at the time of OD event. To our knowledge, this is among the first studies to assess the impact of OATs on ODs in Ontario, Canada.

The demographic characteristics of patients with OUD included in this study were consistent with those from Canadian and international literature. Fatal OD cases were largely among adults aged 35–49 years, whereas non-fatal OD cases occurred more frequently among younger adults aged 18–34 years. The majority of OD cases were male, resided in urban areas, and were disproportionately represented in the lowest income quintile. These findings were consistent with Canadian national data, which reported higher OD burden among males, working-age adults, and socioeconomically disadvantaged populations (6). Globally, OUD prevalence is highest among males aged 20–39 years, with OD mortality risk peaking in the 30–44 age range (19).

In line with prior research, psychiatric comorbidities, particularly anxiety disorders, were highly prevalent among both non-fatal and fatal OD cases (18). The association and trends between anxiety disorders and odds of OD observed in this study highlights the need for integrated models of care that address co-occurring mental health conditions alongside OUD treatment. Other psychiatric comorbidities, including schizophrenia and mood disorders, were also associated with increased odds of OD events, which were consistent with a systematic review linking mental health conditions and opioid-related overdose (19).

Prior literature also reports that factors such as age and polysubstance use may influence the association between treatment patterns and OD risk (20–22). In this study, age and polysubstance use were not included in regression analyses. However, fatal OD cases tended to be older than non-fatal cases. Additionally, a sizable proportion of patients that experienced an opioid-related non-fatal OD concurrently experienced poisoning by narcotics and psychotropic substances. A substantial proportion of fatal OD cases also had non-opioid illicit substances contribute to opioid-related deaths. The patterns of illicit drug use observed in this study in patients with OUD reinforce existing literature that suggests polysubstance use is common in patients with OUD, and is a major contributing factor in OD risk, treatment discontinuation and return to use (23, 24). Taken together, these findings reinforce growing evidence that siloed approaches to treating substance use and mental health conditions may be insufficient for populations with complex clinical needs, and underscore the need for a more integrated care model that accounts for these intersecting risk factors.

Notably, adjusted analyses showed that individuals receiving BUP-XR and TM-BUP were significantly less likely to experience non-fatal and fatal ODs compared with those not receiving OAT, supporting the effectiveness of buprenorphine-based therapies in reducing odds of OD. This was consistent with retrospective cohort studies reporting a lower risk of mortality associated with buprenorphine (11, 25). There were no significant changes in odds of OD observed in methadone compared to no OAT for both fatal and non-fatal OD. This may be attributed to a greater risk of on-treatment OD with methadone compared to buprenorphine (26). Compared to no OAT, SROM was associated with increased odds of OD in both fatal and non-fatal OD cases. This may be attributed to underlying differences in patient characteristics, as SROMs are often only recommended for individuals with severe OUD and those where other OATs are ineffective, contraindicated, or refused (27, 28). The findings of this study may reflect differences in patient populations on different OATs. For example, in this study, OD cases and controls on methadone had the shortest duration of continuous coverage prior to their OD event compared to those on other OATs, which may reflect differences in underlying demographic or clinical characteristics. Future studies with larger sample sizes may further explore demographic and clinical characteristics differences among patients receiving various OATs.

These findings suggest that OAT effects on OD reduction varied by medication type, with buprenorphine-based therapies demonstrating reduced odds of OD, while methadone showed no significant association and SROM was associated with higher odds of OD. The results of this study also support existing Canadian evidence suggesting that early initiation of OAT (inclusive of methadone, buprenorphine-based therapies, and SROM) following hospital discharge is associated with a reduced risk of subsequent opioid toxicity events (hazard ratio = 0.57) (29), while providing a more granular view of the impact of individual OATs on OD risk. Together, these findings reinforce the role of OAT as a cornerstone of OD prevention while highlighting heterogeneity in outcomes across different OAT formulations.

Finally, treatment consistency also emerged as a key contributor to OD risk. Generally, controls in this study often had more sustained and consistent OAT coverage compared with OD cases. Non-fatal OD cases also tended to have more sustained and consistent treatment coverage than fatal cases. Furthermore, the sustained use of BUP-XR and TM-BUP in this study was often longer than that of methadone, accounting for some of the differences in the proportion of observed ODs across those OATs. This finding contrasts with prior studies reporting differences in retention by OAT type, in which methadone is typically associated with longer treatment duration and comparable or superior health outcomes relative to buprenorphine (30–32). Lastly, OAT dosing may be a contributor to treatment retention, and subsequent OD risk. Monthly long-acting injectables like BUP-XR present an important treatment option as they may offer improved adherence, clinical stability at consistent serum concentrations of buprenorphine, and greater convenience with reduced treatment burden compared to daily oral formulations. Rising evidence has shown that monthly BUP-XR may enhance treatment continuity by reducing daily decision-making around medication use and minimizing disruptions related to missed doses or prescription refills, in addition to reducing the risk of relapse (i.e., return to use), ED visits, hospitalizations and detoxification, and better management of comorbidities (33–35). Generally, a greater proportion of non-fatal and fatal OD cases in this study were prescribed low doses of OAT, which supports existing literature that suggests higher doses of OAT may result in greater treatment retention (36, 37). Taken together, these results highlight that consistent OAT coverage is a key factor to reducing OD risk and suggest that buprenorphine may be more successful in reducing OD risk than methadone and SROM in patients with OUD.

This study has several notable strengths. The use of a large, population-based cohort derived from comprehensive administrative health data allowed for robust evaluation of both non-fatal and fatal OD outcomes across multiple OAT modalities. The inclusion of non-fatal OD events provides important insight into earlier points of intervention and risk, complementing findings based solely on mortality data. Furthermore, the study reflects real-world clinical practice in Ontario, providing additional evidence in support of OUD treatment guidelines and policies within the province.

Several limitations should also be acknowledged. First, despite the reliability and widespread use of ICES data, administrative data have inherent limitations, such as potential misclassification due to coding inaccuracies. Specifically, capture of opioid-related non-fatal and fatal OD events and OAT use may be incomplete. Furthermore, as non-fatal ODs were identified based on ED visits and/or hospital discharge records, events that did not result in an ED visit or hospital admission were not captured. Second, in the absence of a direct measure of OAT adherence, patterns of OAT use were reported based on claims data. Third, due to the small sample size of patients who experienced OD events on specific OATs (e.g., 5 or fewer fatal ODs with BUP-XR), demographics and outcomes could not be stratified by index OAT.

Future research should build on these findings by examining the impact of OAT dosing strategies, formulation types (e.g., extended-release vs. daily dosing), and treatment consistency using time-varying analytical approaches. Further investigation into the role of integrated mental health and substance use treatment services may help clarify pathways to reducing odds of OD among individuals with comorbid psychiatric conditions. Qualitative research could offer valuable insights into barriers to OAT retention and adherence, particularly among socioeconomically marginalized populations. Additionally, comparative effectiveness studies across Canadian provinces and evaluations of emerging therapies, including monthly injectable OAT like BUP-XR, may inform national policy development and clinical guideline updates.

## 5 Conclusion

This study demonstrates that the odds of OD while on OATs varies by OAT type and consistency in treatment usage, and that buprenorphine, including BUP-XR and TM-BUP, is associated with a reduced risk of OD among individuals with OUD. Of all OATs, BUP-XR had the lowest proportion of patients with non-fatal and fatal OD compared to no treatment. Furthermore, both BUP-XR and TM-BUP were associated with significantly lower odds of non-fatal and fatal OD compared to no OAT, while methadone and SROM did not show similar improvements. These findings underscore the critical importance of optimizing OAT selection, enhancing treatment retention, and addressing anxiety and comorbid substance use disorders to further mitigate the odds of OD among individuals with OUD. Future research could focus on evaluating dosing strategies, the impact of extended-release formulations, the influence of comorbidities and polysubstance use, and the integration of comprehensive care models to identify barriers to OAT initiation and adherence, especially among disadvantaged populations. These efforts will be essential in shaping evidence-based clinical guidelines and informing policy to improve outcomes for individuals affected by OUD.

## Data Availability

The data analyzed in this study are subject to the following licenses/restrictions: the data supporting these study findings were analyzed by ICES and are not publicly available. Requests to access these datasets should be directed to https://www.ices.on.ca/contact-us/.
