# Supplementary material for: Impact of opioid agonist therapies on non-fatal and fatal overdose: treatment patterns among individuals with opioid use disorder in Ontario, Canada
Source: Front Public Health. 2026 Sep 29;14:1894981. doi: 10.3389/fpubh.2026.1894981 (PMC13657963; doi:10.3389/fpubh.2026.1894981)
Supplement: Supplementary file 1 [file Data_Sheet_1.PDF]

Supplementary Material

1 Supplementary Tables

Supplementary Table 1. Codes used to define opioid use disorder

Data Source	Code(s)
Discharge Abstract Database (DAD), National Ambulatory Care Reporting System (NACRS), Ontario Mental Health Reporting System (OMHRS)	**ICD-10-CA code:**<br>F11: Mental and behavioural disorders due to opioids
Ontario Health Insurance Plan (OHIP), OMHRS	**ICD-9 codes:**<br>3040: Opioid type dependence<br>3047: Combinations of opioid type drug with any other drug dependence<br>3055: Nondependent opioid abuse<br>6483: Drug dependence complicating pregnancy childbirth or the puerperium
OHIP	**OHIP diagnosis code:**<br>304: Drug dependence and drug addiction

Supplementary Table 2. Codes used to identify non-fatal opioid-related overdose

Data Source	Code(s)
DAD, NACRS	**ICD-10-CA codes:**<br>T400: Poisoning by opium<br>T401: Poisoning by heroin<br>T402: Poisoning by other opioids<br>T403: Poisoning by methadone<br>T404: Poisoning by other synthetic narcotics<br>T406: Poisoning by other and unspecified narcotics

Supplementary Table 3. Codes used to identify comorbidities

Variable	Data Source	Code(s)
Human immunodeficiency virus (HIV)	ICES HIV cohort	Identified using ICES-derived validated cohort
Hepatitis C virus (HCV)	DAD, NACRS	**ICD-10-CA codes:**<br>B171: Acute hepatitis C<br>B182: Chronic viral hepatitis C<br>Z2251: Carrier of viral hepatitis C
Substance use disorders	DAD, NACRS, OMHRS	**ICD-10-CA codes:**<br>F10: Mental and behavioural disorders due to use of alcohol<br>F12: Mental and behavioural disorders due to use of cannabinoids<br>F13: Mental and behavioural disorders due to use of sedatives or hypnotics

Variable	Data Source	Code(s)
		F14: Mental and behavioural disorders due to use of cocaine<br>F15: Mental and behavioural disorders due to use of other stimulants, including caffeine<br>F16: Mental and behavioural disorders due to use of hallucinogens<br>F17: Mental and behavioural disorders due to use of tobacco<br>F18: Mental and behavioural disorders due to use of volatile solvents<br>F19: Mental and behavioural disorders due to multiple drug use and use of other psychoactive substances<br>F55: Abuse of non-dependence-producing substances<br><br>**DSM-IV codes (OMHRS):**<br>29100: ALCOHOL - INTOXICATION OR WITHDRAWAL DELIRIUM<br>29110: ALCOHOL - INDUCED PERSISTING AMNESTIC DISORDER<br>29120: ALCOHOL - INDUCED PERSISTING DEMENTIA<br>29130: ALCOHOL - INDUCED PSYCHOTIC DISORDER, WITH HALLUCINATIONS<br>29150: ALCOHOL - INDUCED PSYCHOTIC DISORDER, WITH DELUSIONS<br>29181: ALCOHOL - WITHDRAWAL<br>29182: ALCOHOL - INDUCED SLEEP DISORDER<br>29189: ALCOHOL - INDUCED ANXIETY/MOOD DISORDER OR SEXUAL DYSFUNCTION<br>29190: ALCOHOL - RELATED DISORDER NOS<br>29200: SUBSTANCE - WITHDRAWAL<br>29211: SUBSTANCE - INDUCED PSYCHOTIC DISORDER, WITH DELUSIONS<br>29212: SUBSTANCE - INDUCED PSYCHOTIC DISORDER, WITH HALLUCINATIONS<br>29281: SUBSTANCE - INTOXICATION OR WITHDRAWAL DELIRIUM<br>29282: SUBSTANCE - INDUCED PERSISTING DEMENTIA<br>29283: SUBSTANCE - INDUCED PERSISTING AMNESTIC DISORDER<br>29284: SUBSTANCE - INDUCED MOOD DISORDER<br>29285: SUBSTANCE - INDUCED SLEEP DISORDER<br>29289: SUBSTANCE - INTOXICATION OR INDUCED ANXIETY DISORDER/SEXUAL DYSFUNCTION<br>29290: SUBSTANCE - RELATED DISORDER NOS<br>30300: ALCOHOL INTOXICATION<br>30390: ALCOHOL DEPENDENCE<br>30400: OPIOID DEPENDENCE<br>30410: SEDATIVE, HYPNOTIC, OR ANXIOLYTIC DEPENDENCE<br>30420: COCAINE DEPENDENCE<br>30430: CANNABIS DEPENDENCE<br>30440: AMPHETAMINE DEPENDENCE<br>30450: HALLUCINOGEN DEPENDENCE<br>30460: INHALANT OR PHENCYCLIDINE DEPENDENCE<br>30480: POLYSUBSTANCE DEPENDENCE<br>30490: OTHER (OR UNKNOWN) SUBSTANCE DEPENDENCE<br>30500: ALCOHOL ABUSE<br>30510: NICOTINE DEPENDENCE<br>30520: CANNABIS ABUSE

Variable	Data Source	Code(s)
		30530: HALLUCINOGEN ABUSE<br>30540: SEDATIVE, HYPNOTIC, OR ANXIOLYTIC ABUSE<br>30550: OPIOID ABUSE<br>30560: COCAINE ABUSE<br>30570: AMPHETAMINE ABUSE<br>30590: OTHER (OR UNKNOWN) SUBSTANCE ABUSE
Organic dissociative disorder	DAD, NACRS	**ICD-10-CA code:**<br>F065: Organic dissociative disorder
Mood disorders	DAD, NACRS, OMHRS	**ICD-10-CA codes:**<br>F063: Organic mood [affective] disorders<br>F30: Manic episode<br>F31: Bipolar affective disorder<br>F32: Depressive episode<br>F33: Recurrent depressive disorder<br>F34: Persistent mood [affective] disorders<br>F38: Other mood [affective] disorders<br>F39: Unspecified mood [affective] disorder<br>F530: Mild mental and behavioural disorders associated with the puerperium, not elsewhere classified<br><br>**DSM-IV codes (OMHRS):**<br>29600: BIPOLAR I DISORDER, SINGLE MANIC EPISODE, UNSPECIFIED<br>29601: BIPOLAR I DISORDER, SINGLE MANIC EPISODE, MILD<br>29602: BIPOLAR I DISORDER, SINGLE MANIC EPISODE, MODERATE<br>29603: BIPOLAR I DISORDER, SINGLE MANIC EPISODE, SEVERE WITHOUT PSYCHOTIC FEATURES<br>29604: BIPOLAR I DISORDER, SINGLE MANIC EPISODE, SEVERE WITH PSYCHOTIC FEATURES<br>29605: BIPOLAR I DISORDER, SINGLE MANIC EPISODE, IN PARTIAL REMISSION<br>29606: BIPOLAR I DISORDER, SINGLE MANIC EPISODE, IN FULL REMISSION<br>29620: MAJOR DEPRESSIVE DISORDER, SINGLE EPISODE, UNSPECIFIED<br>29621: MAJOR DEPRESSIVE DISORDER, SINGLE EPISODE, MILD<br>29622: MAJOR DEPRESSIVE DISORDER, SINGLE EPISODE, MODERATE<br>29623: MAJOR DEPRESSIVE DISORDER, SINGLE EPISODE, SEVERE WITHOUT PSYCHOTIC FEATURES<br>29624: MAJOR DEPRESSIVE DISORDER, SINGLE EPISODE, SEVERE WITH PSYCHOTIC FEATURES<br>29625: MAJOR DEPRESSIVE DISORDER, SINGLE EPISODE, IN PARTIAL REMISSION<br>29626: MAJOR DEPRESSIVE DISORDER, SINGLE EPISODE, IN FULL REMISSION<br>29630: MAJOR DEPRESSIVE DISORDER, RECURRENT, UNSPECIFIED<br>29631: MAJOR DEPRESSIVE DISORDER, RECURRENT, MILD<br>29632: MAJOR DEPRESSIVE DISORDER, RECURRENT, MODERATE

Variable	Data Source	Code(s)
		29633: MAJOR DEPRESSIVE DISORDER, RECURRENT, SEVERE WITHOUT PSYCHOTIC FEATURES<br>29634: MAJOR DEPRESSIVE DISORDER, RECURRENT, SEVERE WITH PSYCHOTIC FEATURES<br>29635: MAJOR DEPRESSIVE DISORDER, RECURRENT, IN PARTIAL REMISSION<br>29636: MAJOR DEPRESSIVE DISORDER, RECURRENT, IN FULL REMISSION<br>29640: BIPOLAR I DISORDER, MOST RECENT EPISODE MANIC, HYPO OR UNSPECIFIED<br>29641: BIPOLAR I DISORDER, MOST RECENT EPISODE MANIC, MILD<br>29642: BIPOLAR I DISORDER, MOST RECENT EPISODE MANIC, MODERATE<br>29643: BIPOLAR I DISORDER, MOST RECENT EPISODE MANIC, SEVERE WITHOUT PSYCHOTIC FEATURES<br>29644: BIPOLAR I DISORDER, MOST RECENT EPISODE MANIC, SEVERE WITH PSYCHOTIC FEATURES<br>29645: BIPOLAR I DISORDER, MOST RECENT EPISODE MANIC, IN PARTIAL REMISSION<br>29646: BIPOLAR I DISORDER, MOST RECENT EPISODE MANIC, IN FULL REMISSION<br>29650: BIPOLAR I DISORDER, MOST RECENT EPISODE DEPRESSED, UNSPECIFIED<br>29651: BIPOLAR I DISORDER, MOST RECENT EPISODE DEPRESSED, MILD<br>29652: BIPOLAR I DISORDER, MOST RECENT EPISODE DEPRESSED, MODERATE<br>29653: BIPOLAR I DISORDER, MOST RECENT EPISODE DEPRESSED, SEVERE WITHOUT PSYCHOTIC FEAT<br>29654: BIPOLAR I DISORDER, MOST RECENT EPISODE DEPRESSED, SEVERE WITH PSYCHOTIC FEATURE<br>29655: BIPOLAR I DISORDER, MOST RECENT EPISODE DEPRESSED, IN PARTIAL REMISSION<br>29656: BIPOLAR I DISORDER, MOST RECENT EPISODE DEPRESSED, IN FULL REMISSION<br>29660: BIPOLAR I DISORDER, MOST RECENT EPISODE MIXED, UNSPECIFIED<br>29661: BIPOLAR I DISORDER, MOST RECENT EPISODE MIXED, MILD<br>29662: BIPOLAR I DISORDER, MOST RECENT EPISODE MIXED, MODERATE<br>29663: BIPOLAR I DISORDER, MOST RECENT EPISODE MIXED, SEVERE WITHOUT PSYCHOTIC FEATURES<br>29664: BIPOLAR I DISORDER, MOST RECENT EPISODE MIXED, SEVERE WITH PSYCHOTIC FEATURES<br>29665: BIPOLAR I DISORDER, MOST RECENT EPISODE MIXED, IN PARTIAL REMISSION<br>29666: BIPOLAR I DISORDER, MOST RECENT EPISODE MIXED, IN FULL REMISSION<br>29670: BIPOLAR I DISORDER, MOST RECENT EPISODE UNSPECIFIED<br>29680: BIPOLAR DISORDER NOS<br>29689: BIPOLAR II DISORDER<br>29690: MOOD DISORDER NOS<br>30040: DYSTHYMIC DISORDER<br>30113: CYCLOTHYMIC DISORDER

Variable	Data Source	Code(s)
Anxiety disorders	DAD, NACRS, OMHRS	**ICD-10-CA codes:**<br>F064: Organic anxiety disorder<br>F40: Phobic anxiety disorders<br>F41: Other anxiety disorders<br>F42: Obsessive-compulsive disorder<br>F43: Reaction to severe stress and adjustment disorders<br>F488: Other specified neurotic disorders<br>F489: Neurotic disorder, unspecified<br><br>**DSM-IV codes (OMHRS):**<br>30000: ANXIETY DISORDER NOS<br>30001: PANIC DISORDER WITHOUT AGORAPHOBIA<br>30002: GENERALIZED ANXIETY DISORDER<br>30021: PANIC DISORDER WITH AGORAPHOBIA<br>30022: AGORAPHOBIA WITHOUT HISTORY OF PANIC DISORDER<br>30023: SOCIAL PHOBIA<br>30029: SPECIFIC PHOBIA<br>30030: OBSESSIVE-COMPULSIVE DISORDER<br>30830: ACUTE STRESS DISORDER<br>30900: ADJUSTMENT DISORDER WITH DEPRESSED MOOD<br>30921: SEPARATION ANXIETY DISORDER<br>30924: ADJUSTMENT DISORDER WITH ANXIETY<br>30928: ADJUSTMENT DISORDER WITH MIXED ANXIETY AND DEPRESSED MOOD<br>30930: ADJUSTMENT DISORDER WITH DISTURBANCE OF CONDUCT<br>30940: ADJUSTMENT DISORDER WITH MIXED DISTURBANCE OF EMOTIONS AND CONDUCT<br>30981: POSTTRAUMATIC STRESS DISORDER<br>30990: ADJUSTMENT DISORDER UNSPECIFIED
Post-traumatic stress disorder (PTSD)	DAD, NACRS	**ICD-10-CA code:**<br>F431: Post-traumatic stress disorder
Asthma	ICES ASTHMA cohort	Identified using ICES-derived validated cohort
Chronic obstructive pulmonary disease (COPD)	ICES COPD cohort	Identified using ICES-derived validated cohort
Liver cirrhosis	DAD, NACRS, OHIP	**ICD-10-CA codes:**<br>I859: Oesophageal varices without bleeding<br>I982: Oesophageal varices without bleeding in diseases classified elsewhere<br>K703: Alcoholic cirrhosis of liver<br>K717: Toxic liver disease with fibrosis and cirrhosis of liver<br>K746: Other and unspecified cirrhosis of liver<br><br>**OHIP diagnosis code:**<br>571: Other Diseases of Digestive System: Cirrhosis of the liver (e.g., alcoholic cirrhosis, biliary cirrhosis)
Schizophrenia	DAD, NACRS, OMHRS, OHIP	**ICD-10-CA codes:**<br>F200: Paranoid schizophrenia<br>F201: Hebephrenic schizophrenia<br>F202: Catatonic schizophrenia<br>F203: Undifferentiated schizophrenia<br>F205: Residual schizophrenia

Variable	Data Source	Code(s)
		F206: Simple schizophrenia<br>F208: Other schizophrenia<br>F209: Schizophrenia, unspecified<br>F251: Schizoaffective disorder, depressive type<br>F252: Schizoaffective disorder, mixed type<br>F250: Schizoaffective disorder, manic type<br>F258: Other schizoaffective disorders<br>F259: Schizoaffective disorders, unspecified<br>F29: Unspecified nonorganic psychosis<br><br>**OHIP diagnosis codes:**<br>298: Psychoses: Other psychoses<br>295: Psychoses: Schizophrenia<br><br>**DSM-IV codes (OMHRS):**<br>29510: DISORGANIZED TYPE<br>29520: CATATONIC TYPE<br>29530: PARANOID TYPE<br>29560: RESIDUAL TYPE<br>29570: SCHIZOAFFECTIVE DISORDER<br>29590: UNDIFFERENTIATED TYPE<br>2988: BRIEF PSYCHOTIC DISORDER<br>2989: PSYCHOTIC DISORDER, NOT OTHERWISE SPECIFIED

Supplementary Table 4. ICD-10-CA codes used to identify poisoning by opioids (DAD, NACRS)

Code	Description
T4020	Poisoning by codeine and derivatives
T4040	Poisoning by fentanyl and derivatives
T401	Poisoning by heroin
T4022	Poisoning by hydromorphone
T403	Poisoning by methadone
T4021	Poisoning by morphine
T400	Poisoning by opium
T4023	Poisoning by oxycodone
T4041	Poisoning by tramadol

Supplementary Table 5. ICD-10-CA codes used to identify poisoning by non-opioid illicit drugs (DAD, NACRS)

Code	Description
T51	Poisoning by alcohol
T407	Poisoning by cannabis
T405	Poisoning by cocaine
T408	Poisoning by LSD
T43	Poisoning by psychotropic unclass
T409	Poisoning by unspec psychodysleptics

Supplementary Table 6. ICD-10-CA codes used to identify poisoning by narcotics and psychotropic substances (DAD, NACRS)

Code	Description
X42	Accidental poisoning by narcotics and psychodysleptics
X62	Intentional self-poisoning by narcotics and psychotropic substances
T4028	Poisoning by narc psychodysleptics
T4048	Poisoning by other synth narc unclass
T406	Poisoning by unspec narcotics
Y12	Poisoning of undetermined intent by narcotics and psychotropic substances

Supplementary Table 7. Demographic characteristics - local health integration network

Demographic characteristics	Variable	Base cohort	Opioid-related non-fatal OD cases	Opioid-related non-fatal OD controls	Opioid-related fatal OD cases	Opioid-related fatal OD controls
Local Health Integration Network (LHIN), n (%)	Central	2,521 (5.6%)	198 (4.8%)	2,112 (5.1%)	57 (7.4%)	459 (6.0%)
	Central East	4,199 (9.3%)	400 (9.7%)	3,506 (8.5%)	80 (10.4%)	700 (9.1%)
	Central West	2,098 (4.6%)	98 (2.4%)	1,951 (4.7%)	17 (2.2%)	360 (4.7%)
	Champlain	3,185 (7.0%)	407 (9.9%)	2,868 (7.0%)	75 (9.7%)	530 (6.9%)
	Erie St. Clair	3,805 (8.4%)	217 (5.3%)	3,701 (9.0%)	59 (7.7%)	697 (9.1%)
	Hamilton Niagara Haldimand Brant	6,660 (14.7%)	662 (16.1%)	6,088 (14.8%)	106 (13.8%)	1,183 (15.4%)
	Mississauga Halton	1,511 (3.3%)	148 (3.6%)	1,389 (3.4%)	32 (4.2%)	233 (3.0%)
	North East	4,667 (10.3%)	436 (10.6%)	4,453 (10.8%)	88 (11.4%)	771 (10.0%)
	North Simcoe Muskoka	2,035 (4.5%)	236 (5.7%)	1,723 (4.2%)	36 (4.7%)	328 (4.3%)

Demographic characteristics	Variable	Base cohort	Opioid-related non-fatal OD cases	Opioid-related non-fatal OD controls	Opioid-related fatal OD cases	Opioid-related fatal OD controls
	North West	3,592 (7.9%)	166 (4.0%)	3,758 (9.1%)	34 (4.4%)	635 (8.2%)
	South East	2,068 (4.6%)	245 (6.0%)	1,672 (4.1%)	28 (3.6%)	323 (4.2%)
	South West	3,779 (8.4%)	306 (7.4%)	3,511 (8.5%)	55 (7.1%)	602 (7.8%)
	Toronto Central	3,058 (6.8%)	377 (9.2%)	2,420 (5.9%)	76 (9.9%)	489 (6.4%)
	Waterloo Wellington	2,065 (4.6%)	213 (5.2%)	1,938 (4.7%)	27 (3.5%)	390 (5.1%)

Supplementary Table 8. Presence of illicit substances and drug misuse in opioid-related non-fatal OD cases

Illicit substance use	Opioid-related non-fatal OD cases†, n (%)
	N=4,109
**Poisoning by non-opioid illicit substances**	415 (10.1%)
Poisoning by alcohol	40 (1.0%)
Poisoning by cocaine	224 (5.5%)
Poisoning by cannabis and derivatives	49 (1.2%)
Poisoning by LSD	0 (0.0%)
Poisoning by other and unspecified psychodysleptics (hallucinogens)	*1-5
Poisoning by psychotropic drugs not elsewhere classified	217 (5.3%)
**Poisoning by opioids**	2,866 (69.7%)
Poisoning by opium	13 (0.3%)
Poisoning by heroin	76 (1.8%)
Poisoning by codeine and derivatives	13 (0.3%)
Poisoning by morphine	56 (1.4%)
Poisoning by hydromorphone	71 (1.7%)
Poisoning by oxycodone	60 (1.5%)
Poisoning by methadone	177 (4.3%)
Poisoning by fentanyl and derivatives	2,518 (61.3%)
Poisoning by tramadol	*1-5
**Poisoning by narcotics and psychotropic substances**	4,001 (97.4%)
Poisoning by other opioids not elsewhere classified	985 (24.0%)
Poisoning by other synthetic narcotics not elsewhere classified	47 (1.1%)
Poisoning by other and unspecified narcotics	213 (5.2%)

Illicit substance use	Opioid-related non-fatal OD cases†, n (%)
Intentional self-poisoning by and exposure to narcotics and psychodysleptics (hallucinogens), not elsewhere classified	271 (6.6%)
Poisoning by and exposure to narcotics and psychodysleptics (hallucinogens), not elsewhere classified, undetermined intent	505 (12.3%)
Accidental poisoning by and exposure to narcotics and psychodysleptics (hallucinogens), not elsewhere classified	3,281 (79.8%)

LSD: Lysergic acid diethylamide; OD: Overdose

* Denotes ranges resulting from ICES privacy policies for cell counts are <6. Other counts may also not be disclosed to prevent back-calculation.
† Identified using ICD-10-CA codes.

Supplementary Table 9. Presence of illicit substances and drug misuse in opioid-related fatal OD cases

Illicit substance use	Substances that directly contributed to opioid-related death†, n (%)	Substances detected in opioid-related death‡, n (%)
	N=770	N=770
**Non-opioid substances**	515 (66.9%)	310 (40.3%)
Ethanol	44 (5.7%)	0 (0.0%)
Benzodiazepines	0 (0.0%)	0 (0.0%)
Cocaine	308 (40.0%)	99 (12.9%)
Barbituate (grouped)	0 (0.0%)	0 (0.0%)
Gamma-hydroxybutyrate (GHB)	0 (0.0%)	0 (0.0%)
3,4-Methylenedioxyamphetamine	*1-5	*1-5
3,4-Methylenedioxymethamphetamine	8 (1.0%)	*1-5
Methamphetamine	286 (37.1%)	32 (4.2%)
Methylenedioxypyrovalerone	0 (0.0%)	0 (0.0%)
Illicit Stimulant (grouped) excluding methamphetamines or cocaine	0 (0.0%)	0 (0.0%)
Illicit Stimulant (grouped)	0 (0.0%)	0 (0.0%)
Xylazine	18 (2.3%)	12 (1.6%)
Other drugs (grouped)	*1-5	210 (27.3%)
**Opioid substances**	690 (89.6%)	215 (27.9%)
Acetylfentanyl	11 (1.4%)	9 (1.2%)
Carfentanil	33 (4.3%)	0 (0.0%)
Cyclopropylfentanyl	0 (0.0%)	0 (0.0%)
Despropionylfentanyl	0 (0.0%)	0 (0.0%)
Nitazenes (grouped)	7 (0.9%)	7 (0.9%)

Illicit substance use	Substances that directly contributed to opioid-related death†, n (%)	Substances detected in opioid-related death‡, n (%)
Fentanyl only, does not include fentanyl analogs	664 (86.2%)	*1-5
Fentanyl analogs (grouped)	213 (27.7%)	209 (27.1%)
Total fentanyl and fentanyl analogs (grouped)	681 (88.4%)	*1-5
Furanylfentanyl	0 (0.0%)	0 (0.0%)
Heroin	*1-5	*1-5
Isotonitazene	*1-5	*1-5
Metonitazene	*1-5	*1-5
Para-fluorobutyrylfentanyl	0 (0.0%)	0 (0.0%)
Protonitazene	*1-5	*1-5
Other opioids	6 (0.8%)	0 (0.0%)

* Denotes ranges resulting from ICES privacy policies for cell counts are <6. Other counts may also not be disclosed to prevent back-calculation.
† Substance indicated as “for presentation of substances attributing to death” in DDARD (Drug and Drug/Alcohol-related Deaths) dataset.
‡ Substance indicated as “for presentation of substances detected in death” in DDARD dataset.
